# Beyond immune privilege: the brain as a dynamic immunological interface

**DOI:** 10.1038/s41419-026-08561-z

**Published:** 2026-03-26

**Authors:** Firas Kobeissy, Michel Salzet

**Affiliations:** 1 Department of Neurobiology, Morehouse School of Medicine, Atlanta, GA USA; 2 Univ. Lille, Inserm, CHU Lille, U1192—Protéomique Réponse Inflammatoire Spectrométrie de Masse—PRISM, F-59000 Lille, France; 3 Institut Universitaire de France, Ministère de l’Enseignement supérieur, de la Recherche et de l’Innovation, 1 rue Descartes, 75231, Paris, CEDEX 05 France

**Keywords:** Neuroimmunology, Adaptive immunity

## Abstract

Classically viewed as an “immune‑privileged” site, the central nervous system (CNS) was believed to be shielded from peripheral immune surveillance by the blood–brain barrier (BBB) and the absence of conventional lymphatic vessels. Recent discoveries, particularly the identification of functional meningeal lymphatic vessels and the glymphatic system, recast the CNS as a dynamic immunological interface. Here, we synthesize advances that explain how immune cells access brain border tissues and parenchyma, how resident glia (microglia and astrocytes) shape inflammatory tone and repair, and how gut microbiota, together with regional heterogeneity, refine CNS immunity. We contextualize these mechanisms in disease, including multiple sclerosis, infection, and neurodegeneration, and we outline therapeutic implications that emerge from a revised view of “immune privilege.” This contemporary perspective underscores the importance of targeted CNS immune modulation, aiming to minimize harmful responses while promoting protective mechanisms in the contexts of neuroinflammation, neurodegeneration, and neuro-oncology.

## Facts

- The brain is not immunologically isolated: glymphatic flow and meningeal lymphatics drain CNS antigens/waste to cervical nodes, yet how to **tune** these flows (timing, permeability, route) to slow neurodegeneration or reshape neuroinflammation remains an open, testable frontier.
- The **dural sinus neuroimmune interface**,with constant T-cell surveillance and direct skull bone-marrow inputs,suggests a local, targetable control room for CNS immunity; whether manipulating sinus APC hubs or skull niches can recalibrate disease courses (MS, TBI, glioma) is a pressing question.
- **Compartment rules matter:** meninges, choroid plexus, spinal cord, and parenchyma obey distinct immune logics. Determining if **compartment-targeted** interventions (BBB transport tuning, CSF-route delivery, lymphatic modulation) outperform broad systemic immunotherapy should be a major research program.
- Systemic cues, **microbiota metabolites, sleep, and ageing** shape microglial/astrocytic tone via brain borders. Pinpointing which metabolites reach these interfaces and proving causal, modulable pathways for disease prevention or therapy (AD, PD, MS) is a high-impact, tractable goal.
- The CNS shows **delayed/regulated immune priming** (tolerance checkpoints across the BBB, meninges, and glia). Mapping these checkpoints molecularly and learning to lift or reinforce them **without triggering autoimmunity** could unlock safer grafting, neuro-oncology immunotherapy, and repair after injury.

## The blood–brain barrier (BBB): a dynamic interface

**Fig. 1:**
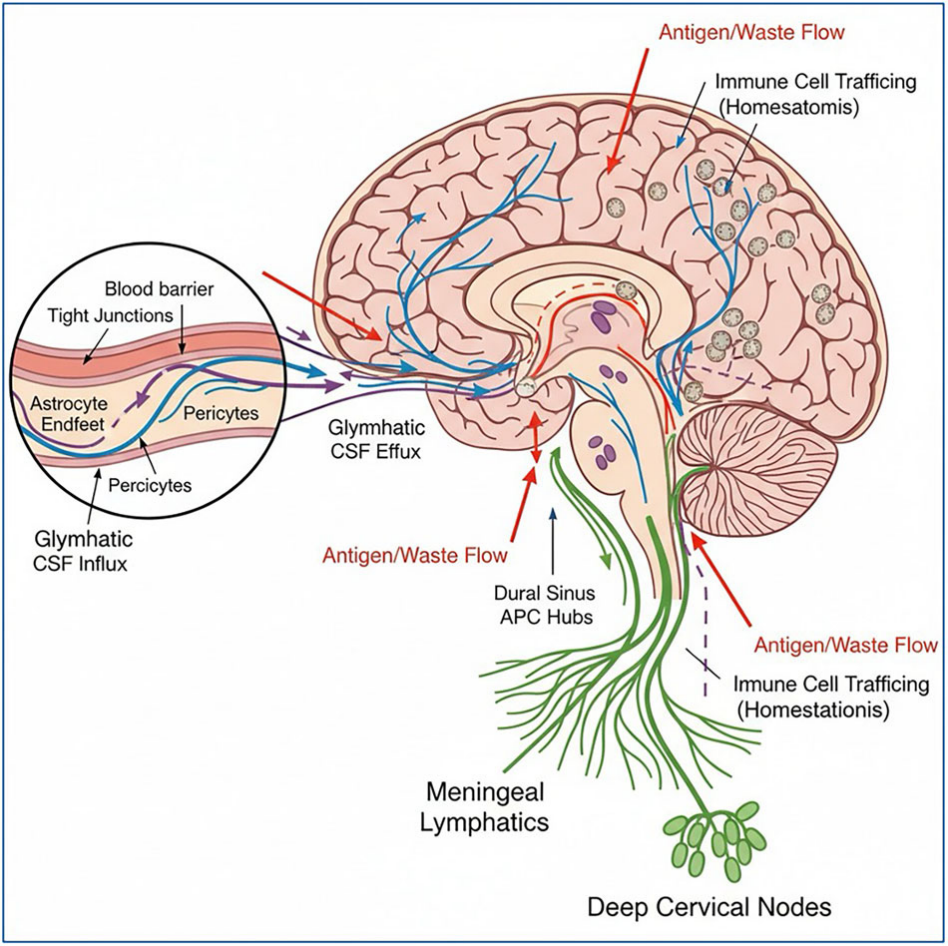
CNS immune interfaces and routes of communication (schematic). The illustration shows BBB (tight junctions, pericytes, astrocyte endfeet), **glymphatic** CSF influx/efflux, **meningeal lymphatics** draining to deep cervical nodes, and **dural sinus APC hubs**. Arrows depict antigen/waste flow and immune cell trafficking under homeostasis vs. inflammation [1–12, 29–32]. (Made with Biorender).

The blood–brain barrier (BBB) has long been regarded as a static, impermeable fortress, serving to shield the CNS from peripheral immune elements. However, recent studies have revealed a more dynamic and selectively permeable nature of the BBB (Table 1). Electron microscopy and advanced imaging techniques have revealed that the BBB consists of densely connected endothelial cells reinforced by astrocyte end-feet and pericyte cells (Fig. 1). This structural intricacy enables the selective entry of immune cells under specific conditions, thereby challenging the prevailing concept of absolute immune exclusion [1]. BBB selective permeability is crucial for maintaining immune homeostasis while allowing for effective immune surveillance to exist [2]. Endothelial cells express adhesion molecules, such as intercellular adhesion molecule-1 (ICAM-1), vascular cell adhesion molecule-1 (VCAM-1), and P-selectin, in response to inflammatory stimuli, facilitating leukocyte adhesion and transmigration. In addition, chemokines, such as CCL2, CXCL10, and CXCL12, secreted by endothelial and glial cells, create chemotactic gradients that guide specific leukocyte subsets into the CNS [3]. This regulated trafficking ensures that immune cells can penetrate the brain when needed, such as during infections or autoimmune diseases, without causing excessive inflammation and tissue damage [4].

**Table 1:** From classical to modern views of CNS immune privilege.

Feature	Classical view	Modern understanding
Blood–brain barrier	Rigid, impermeable barrier	Dynamically regulated; selectively permits leukocyte entry [1]
Lymphatic drainage	Absent in the CNS	**Meningeal lymphatic vessels** drain CSF, linking CNS & peripheral immunity [10, 11]
Resident immune cells	Minimal or inert microglia	**Microglia & astrocytes** actively survey, present antigen, modulate inflammation [16]
Immunological “isolation”	CNS antigens rarely contact peripheral T/B cells	**Glymphatic/lymphatic** egress fosters routine surveillance [7]
Clinical implication	Low graft rejection; non-accessible to immunotherapy	Autoimmunity and immunotherapy responses demonstrate access [56**–**58]

Of interest, the recent discovery of the glymphatic system has further elucidated the mechanisms of waste clearance and immune surveillance in the CNS [5]. The glymphatic system partly depends on astrocyte aquaporin-4 channels to enable the convective flow of cerebrospinal fluid (CSF) and the clearance of interstitial solutes along the peri-arterial spaces [6, 7]. Operating predominantly during sleep, the glymphatic system facilitates the clearance of metabolic “toxic” waste products and misfolded proteins, including tau/ptau and beta-amyloid proteins. While not an immunological pathway per se, the glymphatic system intersects with immune processes by handling debris that may include antigenic molecules. Inefficiencies, blockages or dysregulation of this clearance system would alter the immune environment of the CNS, influencing microglial and peripheral immune cell responses [5]. Research has demonstrated that the glymphatic system is particularly active during sleep, with the CSF flow increasing by up to 60% compared to awake states [8]. This enhanced flow is crucial for the clearance of tau and beta-amyloid proteins implicated in Alzheimer’s disease (AD) [9]. Research by Iliff et al. [7] demonstrated that the glymphatic system is responsible for the clearance of approximately 70% of beta-amyloid from the brain, highlighting its significance in maintaining CNS homeostasis [7]. The glymphatic system’s efficiency is also influenced by aging, sleep disorders and other conditions such as neurodegenerative diseases (AD and brain trauma), which can impair its function and contribute to the accumulation of toxic proteins in the brain [9]. The clearance pathway is thus crucial for the initiation of adaptive immune responses, as it enables the presentation of CNS-derived antigens to T cells in the lymph nodes, consequently leading to the activation and differentiation of antigen-specific T cells. These activated T cells can then migrate back to the CNS, crossing the BBB to mount immune responses [10]. Thus, the BBB functions as a dynamic and adaptable interface that balances protection and permissiveness; consequently, enabling the CNS to maintain its specialized environment while allowing necessary immune interactions [4].

## Meningeal lymphatic vessels: a paradigm shift

The discovery of functional lymphatic vessels in the dura mater in 2015 marked a significant paradigm shift in our understanding of CNS immune surveillance [10, 11]. Two independent studies have revealed the presence of lymphatic channels within the meninges, which are capable of draining CSF and interstitial fluid into the deep cervical lymph nodes (Fig. 1). The identification of these meningeal lymphatics was achieved by the use of classical lymphatic markers, including the Lyve-1, Prox1, and VEGFR3 protein markers. The anatomical orientation of these lymphatics, which run parallel to the venous sinuses, suggests a potential anatomical route for the egress of the CNS antigens [12]. This finding necessitates the reevaluation of the CNS interaction with the peripheral immune system. It highlights a previously unrecognized link that facilitates immune surveillance and response [13, 14]. Meningial lymphatic vessels occurrence challenges the long-standing concept of the CNS as an immune-privileged site and underscores the dynamic interplay between the CNS and the peripheral immune system. This drainage pathway ensures that molecules from the CNS parenchyma can reach peripheral lymphoid tissues, thereby linking local and systemic immune processes. This integrated immune network facilitates both an effective immune surveillance and response, while maintaining the delicate balance necessary to prevent excessive neuroinflammation and tissue damage in the CNS. The existence and functionality of meningeal lymphatic vessels have been demonstrated by compelling evidence provided by Louveau et al. (2015) and Aspelund et al. [10, 11]. Utilizing state-of-the-art imaging methodologies, it was established that these vessels enable the drainage of CSF, interstitial fluid, immune cells, and antigens from the CNS to the deep cervical lymph nodes. This discovery has also paved the way for novel research avenues that explore the functional roles of these vessels in both health and disease. Consequently, future research endeavors are expected to explore the therapeutic potential of modulating meningeal lymphatic function for the treatment of neuroinflammatory and neurodegenerative diseases [15].

## How immune cells enter and move within the CNS

**Fig. 2:**
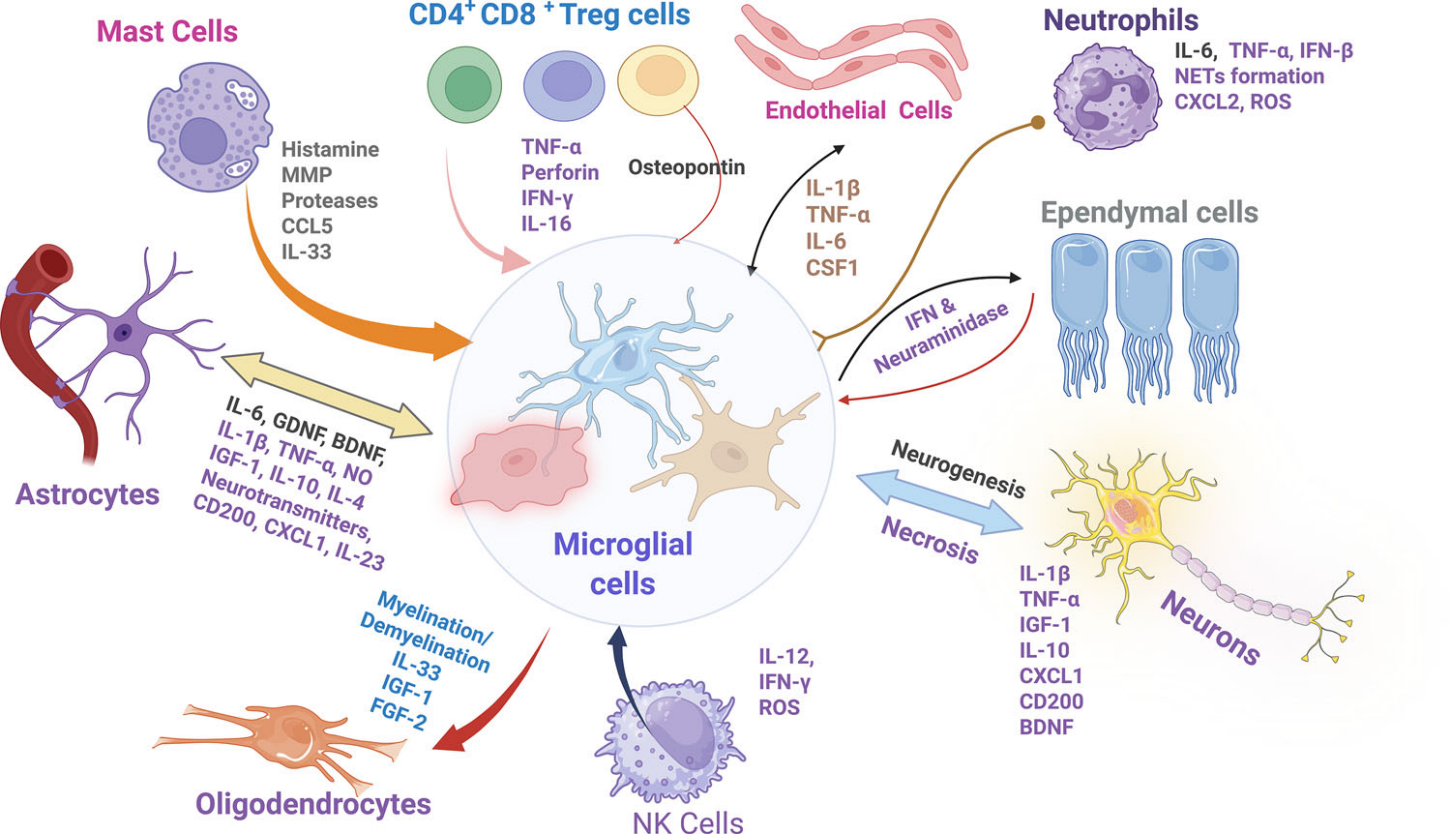
Microglia-centered crosstalk in the CNS. Schematic of bidirectional interactions between microglia (context-dependent functional states) and neighboring neural, glial, endothelial and infiltrating immune cells. Astrocytes release IL-6, GDNF, BDNF, IL-1β, TNF-α, nitric oxide, IL-17, IL-10, IL-4, neurotransmitters, CD200, CXCL1, and IL-23 to shape microglial activation. Oligodendrocytes provide myelination/demyelination cues and growth factors, including IL-33, IGF-1, and FGF-2. Mast cells influence microglia via histamine, matrix metalloproteinases, proteases, CCL5 and IL-33. CD4⁺, CD8⁺, and Treg lymphocytes deliver TNF-α, perforin, IFN-γ and IL-16, with osteopontin contributing to microglia–lymphocyte crosstalk. Endothelial cells supply IL-1β, TNF-α, IL-6, and CSF1 that promote recruitment and survival. Neutrophils communicate through IL-6, TNF-α, and IFN-β, generate neutrophil extracellular traps, secrete CXCL2 and release reactive oxygen species. Ependymal cells signal through interferons and neuraminidase. Neurons modulate microglia with IL-1β, TNF-α, IGF-1, IL-10, CXCL1, CD200, and BDNF and are, in turn, affected along a continuum from neurogenesis to necrosis. Microglia also engage dendritic cells and natural killer cells via IL-12, IFN-γ, and reactive oxygen species, collectively orchestrating protective or damaging responses within the neural parenchyma. Arrows denote the predominant direction of influence; listed mediators are representative rather than exhaustive. (Made with Biorender).

Microglia, the primary resident immune cells in the CNS, play a pivotal role in immune surveillance and response [16]. Derived from yolk-sac progenitors, microglia populate the developing CNS and remain there throughout the organism’s lifetime. Microglia are continuously active and react to the stage of life, CNS region, species, sex, and context of health or disease by adopting different states and performing different functions [17]. At baseline, microglia are highly motile, continuously extending and retracting their processes to continuously monitor local signals. Upon stimulation by infection, tissue damage, or disease-related pathological conditions, microglia can adopt distinct activation states, producing a context-dependent repertoire of inflammatory and regulatory mediators [16]. Through their phenotypic plasticity, microglia shape local immune responses and guide peripheral immune cell infiltration [16]. Furthermore, microglia’s ability to adopt antigen-presenting cell (APC)-like functions further challenges the notion of CNS immune privilege. Microglia can upregulate MHC class II molecules and present antigens to CD4+ T cells, initiating adaptive immune responses within the CNS [16]. This capacity for antigen presentation, along with the discovery of meningeal lymphatic vessels, both provide a clear path for CNS-derived antigens to reach peripheral lymphoid tissues, where adaptive immune responses can be mounted [18]. Along the same line, astrocytes, the most numerous glial cells in the CNS, also contribute to immune modulation. Although primarily involved in metabolic support and ion homeostasis, astrocytes undergo reactive changes in response to trauma, infection, or chronic disease. In such states, astrocytes produce chemokines and cytokines that regulate immune cell recruitment and modulate microglial function. This immunomodulatory role places astrocytes at the intersection of neuroprotection and neuroinflammation, further emphasizing the intrinsic immune competence of the CNS [19]. Experimental evidence has shown that astrocytes can produce a variety of cytokines (interleukins-IL) and chemokines, including IL-1β, IL-6, TNF-α, and CCL2 [20] (Fig. 2).

Interleukin and chemokine molecules play crucial roles in recruiting immune cells to sites of injury or infection and modulating their activity (Table 2). For instance, IL-1β and TNF-α, which are secreted by astrocytes, can activate microglia, thereby enhancing their phagocytic activity and cytokine production. Conversely, astrocytes can also produce anti-inflammatory cytokines like IL-10 and TGF-β, which act as anti-inflammatory molecules and promote tissue repair. This dual role of astrocyte function in both initiating and resolving inflammation highlights their importance in maintaining CNS immune homeostasis. Moreover, astrocytes have been shown to play a critical role in the formation and maintenance of the BBB. They express a variety of molecules that contribute to the maintenance of tight junctions between endothelial cells, including claudin and occludin proteins [21]. Astrocytes also produce factors that regulate the expression of adhesion molecules and chemokines by endothelial cells, thereby influencing the trafficking of homing immune cells into the CNS. This regulatory role further underscores the integral role of astrocytes in CNS immune surveillance and response [22].

**Table 2:** Context-dependent functions of key cytokines in the CNS (examples).

Mediator	Canonical roles	Context-dependent effects (illustrative)	Refs.
IL-1β	Pro-inflammatory; recruits leukocytes	Can enable repair via microglial phagocytosis in controlled settings; excess drives neurotoxicity	[16, 20, 42]
TNF-α	Pro-inflammatory; BBB activation	Bidirectional: promotes demyelination vs. supports remyelination depending on receptor/context	[16, 20, 48, 51]
IL-6	Acute phase signaling	Protective vs. detrimental depending on timing/compartment	[20, 51]
IFN-γ	Antiviral, macrophage activation	Modulates social behavior/synaptic pruning in specific contexts	[29, 51]
IL-10	Anti-inflammatory, limits damage	Over-suppression may impair pathogen/aggregate clearance	[42, 43]
TGF-β	Immune regulation; Treg induction	Excess may hinder repair; essential for tolerance	

Nevertheless, our understanding of how the brain and immune system interact has changed substantially over the past decades. Initially, the brain was thought to be immunoprecipitated and isolated from the rest of the body. Recent discoveries such as the role of tissue-resident microglia in CNS immune surveillance and the existence of a complex brain–immune network involving peripheral immune players highlight the intricate cross-talk underlying brain–immune interactions [23, 24]. This complex brain–immune relationship is important during development, adulthood, and ageing, as well as during various pathologies. Research on graft survival in the CNS has undergone reevaluation, revealing that many CNS grafts are not spared from immune-mediated destruction but rather experience delayed rejection [25**–**27]. This delayed rejection is attributed to the regulated nature of leukocyte entry, the limited number of local professional antigen-presenting cells, and a baseline immunosuppressive cytokine milieu [25**–**27]. As vascularization of the graft occurs and antigens begin to drain into the cervical lymph nodes, T cells and other effector cells are eventually primed, leading to graft rejection. These findings indicate that the CNS represents a state of regulated, low-level immune reactivity under baseline conditions, rather than an absolute or permanent immune-privileged environment [28]. The delayed kinetics of CNS immune responses are now understood to result from low baseline expression of adhesion molecules, the scarcity of local dendritic cells, and restricted capacity for costimulation. However, once inflammatory cascades are initiated, the CNS morphs into a site of vigorous immune activity comparable to peripheral tissues. Thus, rethinking brain immunity could reveal new therapeutic targets for various neurological disorders (Boxes 1 and 2).

The clearance of brain interstitial fluid into the surrounding CSF revealed a potential pathway facilitating CNS immune surveillance. This flow mechanism shuttles the soluble immunogenic constituents of the brain, including microbial pathogens, tumor neoantigens, and even self-antigens, out of the CNS and enables access to peripheral tissues. We therefore reasoned that the detection of these soluble antigens could allow the immune system to continuously probe for potential CNS dysfunction and explore the locations and mechanisms mediating such monitoring [29, 30]. Neuroimmune surveillance occurs primarily at the edges of the CNS, in brain–border tissues called the dural meninges [31]. Instead of CSF flowing into venous blood, MR imaging data revealed that CNS-derived antigens in the CSF rapidly accumulated in the dural meninges, specifically surrounding large vascular structures called the dural sinuses [8, 29, 31]. Demonstrating evolutionary conserved fluid dynamics, this pathway was also visualized by magnetic resonance imaging (MRI) in human subjects, while a liquid chromatography mass spectrometry (LC–MS) study revealed CNS-enriched antigens surrounding human dural sinuses [31].

Critically, the dural sinuses also contain highly organized hubs of antigen-presenting cells capable of capturing brain-derived antigens and presenting them on their surface for recognition by another specialized immune cell, the T cell. All these components are concentrated at the dural sinuses; However, a key question remains: whether adaptive immune cells are capable of surveilling this tissue. A single-cell RNA sequencing (scRNA-seq) meningeal atlas has been generated indicated that the dural sinuses contain a stromal niche perfectly primed for T cell extravasation *via* specialized endothelium and stromal-derived chemokines [32]. Besides, using in vivo imaging and transfer of antigen-specific T cells studies, it was shown that, unlike the CNS vasculature, T cells constantly infiltrate this site and, upon recognition of CSF-derived antigens, they initiate an effector response designed to restore brain homeostasis [29, 31]. Also, overlying the dural sinuses, a large pool of bone marrow niche is identified, situated on the periphery of the brain. The formation of new blood cells occurs via resident lymphoid and myeloid progenitors. In mice and humans, this bone marrow niche is directly connected to the underlying dura mater by way of ossified channels, providing direct and uninterrupted passage between the bone marrow of the skull and the underlying dural meninges. Parabiotic pairing (parabiosis), skull transplantation models, and selective irradiation of discrete bone marrow niches were assessed in mice to demonstrate that these channels are conduits for homeostatic myeloid and lymphoid cell trafficking from the skull and vertebral bone marrow [30, 33]. These vascular highways allow B cells, neutrophils, and monocytes to make their way into the underlying dural tissue and provide substantial contributions to its immune repertoire, wholly independent of a blood route. Such a pathway allows for the delivery of a particular subset of immune cells directly to the CNS border tissues. Neuroimmune surveillance at the dural sinuses is hypothesized to detect CNS dysfunction and relay it to adjacent bone marrow, which in turn deploys immune cells to address various insults [34]. For example, post-neuroinflammatory injury induced by experimental autoimmune encephalomyelitis (EAE), a mouse model of multiple sclerosis, showed elevated numbers of CNS bone marrow-derived cells present in the dural meninges and no longer restricted to the CNS borders. Furthermore, Monocytes infiltrating the CNS were primarily of local bone marrow origin and frequently associated with bone marrow channels. Similar patterns were observed after optic nerve and spinal cord injuries, demonstrating a conserved ability of CNS-associated bone marrow-derived cells to infiltrate CNS tissue in the wake of varied pathologies [35**–**39]. Not only does this local bone marrow niche function as an immunological reservoir, but the cells originating from this niche are phenotypically distinct. Cells originating from local bone marrow likely play distinct roles in CNS injury, shaped by their specific ontogeny and interactions with stromal niches during transit through the dura [39]. The complexities of neuroimmune-cytokine-chemokine interactions are continuously being unraveled.

Endothelial cells in the CNS selectively express several molecules, including ICAM-1, VCAM-1, and P-selectin, in response to inflammatory cues. These changes, coupled with the secretion of chemotactic cues including CCL2, CXCL10, and CXCL12, guide different subsets of leukocytes across the barrier [40, 41]. Glial cells orchestrate much of this process by producing and responding to cytokines like IL-1β, IL-6, tumor necrosis factor (TNF-α), and type I interferons. The result is a constantly modulated environment in which traffic into and out of the CNS can be upregulated or suppressed depending on the microenvironment demands. Meningeal lymphatic vessels, now recognized as an essential drainage pathway, ensure that molecules from the CNS parenchyma can reach peripheral lymphoid tissues for antigen presentation, thereby linking local and systemic immune processes. Several regulatory mechanisms are in place to maintain immune homeostasis in the CNS. These include the expression of immunosuppressive cytokines such as IL-10 and TGF-β, the presence of regulatory T cells (Tregs), and the limited expression of co-stimulatory molecules on antigen-presenting cells (APCs). These complex mechanisms regulate the extent and duration of immune responses, preventing excessive inflammation and tissue damage. Studies have shown that IL-10 and TGF-β play essential roles in suppressing inflammatory responses in the CNS. For instance, IL-10 produced by microglia and astrocytes can inhibit the production of pro-inflammatory cytokines by activated immune cells, thereby limiting inflammation and tissue damage [42]. Similarly, TGF-β produced by astrocytes can promote the differentiation of regulatory T cells, which help suppress immune responses and maintain immune tolerance in the CNS. Furthermore, Tregs are thought to play a crucial role in preventing autoimmune responses and maintaining immune tolerance and homeostasis in the CNS [43]. Taken together, these findings have uncovered an immunological orchestration that permits the CNS to maintain its barriers and enable tight regulation of cerebral functions while still allowing antigenic sampling to recognize CNS perturbations. Through this clever compromise, the CNS confines immunity to its borders, preserving neuronal function while maintaining immunological protection. These findings of novel paradigms have reshaped our understanding of neuroimmune surveillance.

Along the same line, the gut-brain axis represents a bidirectional communication network between the gastrointestinal tract and the CNS, significantly influencing neuroimmune interactions orchestrated by the gut microbiota [44]. Studies on gut microbiota have been linked to central immunological changes impacting a spectrum of neurological conditions, including autism spectrum disorders, Parkinson’s disease, and multiple sclerosis [44, 45]. Gut microbiota-derived metabolites, such as short-chain fatty acids (SCFAs), enter the systemic circulation and can modulate peripheral immune cell differentiation [44]. These metabolites may cross a permissive BBB or indirectly influence CNS immune responses by altering peripheral immune cell populations [45]. For instance, SCFAs have been shown to enhance the differentiation of Tregs [45]. Animal studies show that germ-free mice exhibit microglial defects in maturation and reactivity, demonstrating that the immune environment of the brain depends on signals that originate outside of what was once considered a fully secluded region [45]. These findings indicate that the CNS immune environment is intricately shaped by systemic factors extending beyond its traditional boundaries [45].

The interplay between the gut microbiota and regional CNS immunity is further evidenced by studies linking gut dysbiosis to exacerbated neuroinflammation and neurodegeneration [44]. Dysbiotic gut microbiota can lead to increased systemic inflammation, which in turn amplifies CNS immune responses [45]. This bidirectional relationship underscores the importance of maintaining a balanced gut microbiome for CNS immune homeostasis [44]. Understanding these interactions is crucial for developing targeted therapies that address specific compartments of CNS immunity [23]. Therapeutic strategies modulating gut microbiota, such as probiotics, prebiotics, and dietary interventions, hold promise for influencing CNS immune responses and ameliorating neurological disorders [44].

To add to the Gut-brain axis, regional heterogeneity within the CNS further complicates the immune landscape [22, 46]. The blood-spinal cord barrier, for example, exhibits similar structural features to the BBB but responds differently to local and systemic inflammatory stimuli [22, 46]. The choroid plexus forms a specialized blood-CSF barrier with fenestrated capillaries and epithelial cells that demonstrate distinct immunological activity [24]. While the meninges are rich in immune cells and host the newly discovered lymphatic vessels [10], the CNS parenchyma maintains a more restrictive immune environment, primarily inhabited by microglia [16]. These distinct immune zones contribute to the spatial regulation of immune responses, influencing the susceptibility of specific CNS regions to immune infiltration and inflammation [24]. Moreover, different brain regions exhibit varying degrees of immune cell infiltration and activation in response to injury or infection [47]. For example, the hippocampus and cortex display distinct patterns of microglial reactivity and cytokine production following ischemic stroke, with the hippocampus showing more pronounced inflammatory responses [47]. Similarly, in Parkinson’s disease, the substantia nigra and striatum brain regions demonstrate differential immune responses [47]. The gut-microbiota impacts this regional heterogeneity and the immune response. The interplay between the gut microbiota and regional CNS immunity is further evidenced by studies linking gut dysbiosis to exacerbated neuroinflammation and neurodegeneration [44]. Dysbiotic gut microbiota can lead to increased systemic inflammation, which in turn amplifies CNS immune responses [45]. This bidirectional relationship underscores the importance of maintaining a balanced gut microbiome for CNS immune homeostasis [44]. Understanding these interactions is crucial for developing targeted therapies that address specific compartments of CNS immunity [23]. Therapeutic strategies that modulate the gut microbiota, such as probiotics, prebiotics, and dietary interventions, hold promise for influencing CNS immune responses and ameliorating neurological disorders [44]. Thus, the gut-brain axis introduces an additional layer of complexity to CNS immune regulation. Regional heterogeneity within the CNS further necessitates nuanced approaches to understanding and modulating CNS immunity for therapeutic benefit. Collectively, these insights show that the gut–brain axis adds complexity to CNS immune regulation, wherein regional heterogeneity and gut microbiota provide novel opportunities to understand and therapeutically modulate CNS immunity.

Box 1 Key takeaways

- The CNS is not immunologically isolated: meningeal lymphatics and the glymphatic system couple brain antigen/waste drainage to peripheral immune organs [7, 10, 11].
- Brain borders (dura/meninges, choroid plexus) concentrate surveillance, while parenchyma is guarded by regulated entry across the BBB and by resident glia [1–4, 16, 22, 31].
- Microglial and astrocyte reactivity is context‑dependent, producing both protective and deleterious outcomes; anti‑inflammatory circuits (e.g., IL‑10/TGF‑β, Tregs) constrain damage [16, 20, 42, 43].
- Systemic cues (microbiota metabolites, aging, sleep) shape CNS immune tone via BBB and border interfaces [5, 8, 22, 44, 45].
- Emerging therapies increasingly target interfaces and compartments (BBB, meninges, CSF flow) to balance repair vs. injury in MS, trauma, degeneration, and brain tumors [56**–**59, 61, 62].

Box 2 Outstanding questions

- Regulating meningeal lymphatics: How do neural/systemic signals control permeability and remodeling, and can these be leveraged therapeutically? [10, 11, 15]
- CNS‑specific tolerance: Why are CNS antigen responses delayed? Which meningeal or microglial checkpoints mediate this tolerance?
- Gut–brain modulation: Which microbial metabolites reach the brain/borders to influence microglial reactivity, and can microbiota modulation prevent neuroinflammation? [23, 24, 44, 45]
- Compartment‑targeted therapy: Given heterogeneity (meninges vs. parenchyma vs. spinal cord), will region‑specific interventions outperform systemic approaches? [24, 56]
- Aging & glymphatic decline: Does reduced flow accelerate neurodegeneration, and can CSF clearance be boosted to slow disease? [5, 7, 8]

## Pathological conditions and immune responses

**Fig. 3:**
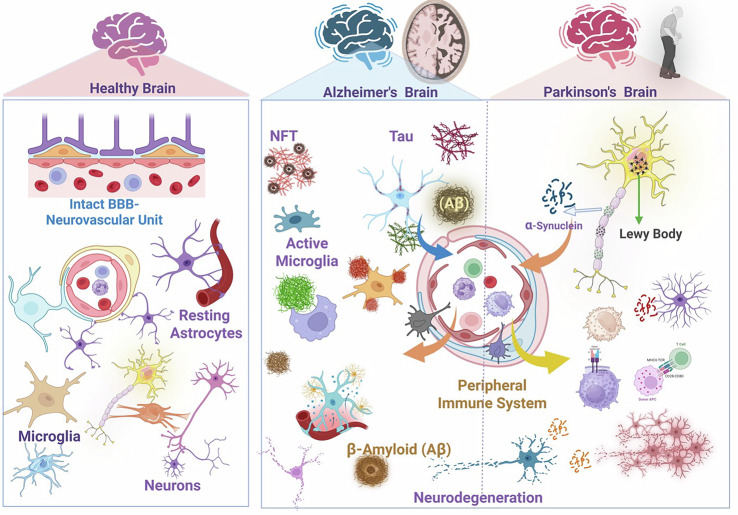
Neuroimmune interactions in health and neurodegeneration. In the healthy brain, neurons, astrocytes, microglia, and endothelial cells within the neurovascular unit preserve homeostasis and restrict immune cell entry. In Alzheimer’s disease, amyloid-β and tau aggregates drive microglial activation and neuroinflammation, whereas in Parkinson’s disease, α-synuclein accumulation induces astrocyte reactivity. Both conditions enhance communication with the peripheral immune system, promoting chronic neuroinflammation and disease progression. (made with Biorender).

Despite the brain’s heightened regulatory environment, numerous pathological conditions demonstrate the occurrence of potent immune responses within the CNS. Multiple sclerosis serves as a paradigmatic example, where autoreactive T cells become primed against myelin components in peripheral lymphoid organs, cross the BBB, and trigger demyelination. Activated microglia [47] and macrophages [48] contribute to myelin destruction, while cytokines, chemokines, and complement factors propagate lesions [47, 48]. This autoimmune attack provides unequivocal evidence that the CNS is not an untouchable immune sanctuary. Infectious diseases of the CNS, detected in meningitis, encephalitis, and brain abscesses, further illustrate the robust immune responses that can occur within the brain [49]. Pathogens can breach or bypass the BBB, eliciting a strong inflammatory response characterized by microglial reactivity, recruitment of neutrophils and monocytes, and extensive cytokine production [49]. HIV infection, for example, can lead to HIV-associated neurocognitive disorders through chronic immune activation involving resident glia and infiltrating lymphocytes [49]. In cerebral malaria, parasites sequester in the cerebral microvasculature, triggering a cascade of endothelial dysfunction and inflammation that results in severe neurological complications [50]. Neurodegenerative diseases have also increasingly been recognized for their inflammatory components [51]. In Alzheimer’s disease, microglia surround amyloid-beta plaques, releasing inflammatory mediators and participating in plaque clearance or exacerbation [52]. Genetic studies have identified microglial genes, such as TREM2, that can influence disease risk and progression [53]. Studies using transgenic mouse models of Alzheimer’s disease have demonstrated that microglia can phagocytose and degrade amyloid-beta, thereby reducing plaque burden and improving cognitive function [54]. However, microglia can also contribute to neuroinflammation and neurodegeneration by producing pro-inflammatory cytokines and reactive oxygen species. The balance between microglial phagocytic activity and inflammatory responses is critical for determining the outcome of Alzheimer’s disease pathology. Furthermore, studies have shown that microglia can adopt different phenotypes depending on the microenvironment and the stage of the disease. In the early stages of Alzheimer’s disease, microglia can assume an anti-inflammatory phenotype, characterized by the production of anti-inflammatory cytokines and the promotion of tissue repair. However, as the disease progresses, they transition to a pro-inflammatory phenotype, producing cytokines that exacerbate neuroinflammation and neurodegeneration [55] (Fig. 3).

**Fig. 4:**
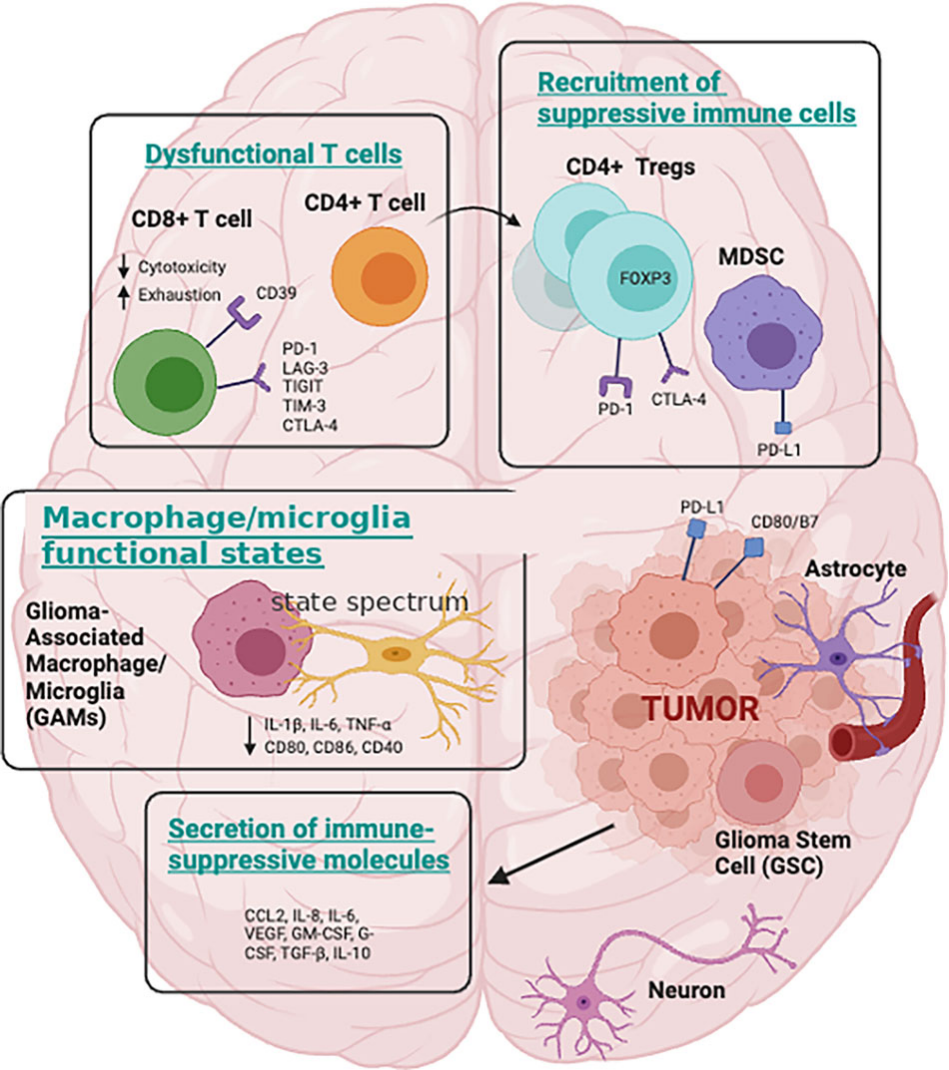
Mechanisms of immune evasion in the glioma microenvironment. Glioma progression is sustained by multiple immunosuppressive mechanisms. Dysfunctional CD8⁺ and CD4⁺ T cells exhibit exhaustion and reduced cytotoxicity through upregulation of checkpoint receptors (PD-1, LAG-3, TIGIT, TIM-3, CTLA-4). Tumors recruit suppressive immune cells, including FOXP3⁺ regulatory T cells and myeloid-derived suppressor cells (MDSCs), which express PD-1/PD-L1 and CTLA-4. Glioma-associated macrophages and microglia (GAMs) adopt tumor-associated, immunosuppressive activation states, releasing pro-tumorigenic cytokines (IL-1β, IL-6, TNF-α) while downregulating costimulatory molecules (CD80, CD86, CD40). In parallel, glioma and stromal cells secrete immunosuppressive mediators (CCL2, IL-8, IL-6, VEGF, GM-CSF, TGF-β, IL-10) that further dampen anti-tumor immunity. Together, these interactions create a permissive niche for glioma stem cells and tumor expansion within the CNS. (Made with Biorender).

In multiple sclerosis, therapeutic agents including natalizumab and fingolimod effectively limit pathological immune cell infiltration into the CNS, thereby reducing disease severity [55**–**57]. B-cell depletion therapies, such as ocrelizumab, also demonstrate marked clinical efficacy in MS, highlighting the crucial role of adaptive immune mechanisms in CNS disease [56**–**58]. In cancer treatment, checkpoint inhibitors and chimeric antigen receptor (CAR) T cells have shown promise in controlling specific brain metastases, illustrating the CNS’s responsiveness to systemic immunomodulatory interventions [59]. Clinically, moving beyond the immune-privileged model carries important implications for the development of therapies targeting autoimmune, autoinflammatory, and neurodegenerative disorders. Understanding that the CNS is highly susceptible to immune-mediated damage has prompted the development of therapies designed to selectively restrict pathological immune cell entry or function. For neurodegenerative conditions, strategies to harness or fine-tune immune responses, potentially aiding in the clearance of protein aggregates, may represent new therapeutic avenues. In oncology, immunotherapies that mobilize T cells or direct them to brain tumors illustrate how the CNS environment can be manipulated to enhance anti-tumor immunity [59**–**62] (Fig. 4). The clinical implications of these findings are far-reaching. TILs are thought to play a crucial role in recognizing and eliminating tumor cells, and their presence is often correlated with better clinical outcomes [60].

## Contemporary vision and future directions

The contemporary vision of CNS immunity has evolved from a simplistic view of immune privilege to a sophisticated appreciation of a dynamic and immunologically active environment (Table 1). The CNS is now recognized as a site of finely calibrated immunological engagement, where resident immune cells, regulated barrier properties, and newly discovered lymphatic structures participate in a larger, deeply integrated system that operates across the entire body. This unifying lesson highlights the critical importance of elucidating the mechanisms of CNS immune regulation to improve therapies for neurological disorders.

A deeper understanding of CNS immune regulation will enable the refinement of therapies for multiple sclerosis, Alzheimer’s disease, Parkinson’s disease, stroke, brain tumors, and numerous other CNS disorders. One of the foremost challenges in translational neuroscience and immunology is defining how to modulate inflammatory and reparative brain processes. Current strategies seek a balance between leveraging the immune system’s protective and clearing functions while minimizing the risk of permanent neural injury. However, significant limitations remain. First, many current insights into CNS immunity are based and derive from rodent models; however, interspecies differences in barrier permeability, microglial reactivity, and meningeal lymphatic function constrain direct applicability to humans. Second, the dual and sometimes opposing roles of cytokines such as TNF-α, IL-6, and IFN-γ create challenges in designing therapies that avoid tipping the balance toward pathology. Third, restricted penetration of the BBB limits the effectiveness of most immunomodulatory drugs, often requiring local delivery or systemic dosing strategies that carry significant risks of adverse effects. Finally, several clinical trials of neuroimmune modulation (e.g., anti-TNF in multiple sclerosis, checkpoint blockade in gliomas) have yielded disappointing or variable outcomes, highlighting the need for more precise patient stratification and biomarkers of CNS immune states.

Accordingly, the once-dominant concept of “immune privilege” has evolved into a nuanced recognition of the CNS as a site of precisely regulated immune activity, distinct from but not isolated relative to other tissues. The unifying lesson is that every aspect of the CNS immune environment, from barrier properties to resident glia to newly discovered lymphatic structures, participates in a larger, deeply integrated system that operates across the entire body. Current debates revolve more around how to harness or optimize CNS immune responses for therapeutic benefit rather than whether the brain can initiate or accommodate immune events. For multiple sclerosis and other inflammatory demyelinating conditions, the clinical community continues to test new immunomodulatory agents designed to specifically inhibit pathogenic subsets of T or B cells, or to block cytokine axes. In stroke and traumatic brain injury, research aims to modulate the post-injury inflammatory phase to promote tissue repair while mitigating secondary damage caused by excessive leukocyte infiltration. In the context of neurodegenerative disease, current efforts focus on promoting beneficial microglial phagocytosis and peripheral macrophage infiltration for protein aggregate clearance, while minimizing the risk of damaging inflammatory cascades. Collectively, these strategies reflect the premise that the CNS is accessible to immune cells and capable of directing local immune processes.

## Conclusion

The conceptual evolution of immune privilege within the CNS underscores the dynamic nature of scientific paradigms. Initially based on observations such as graft survival studies, the robust BBB, and the presumed absence of lymphatic channels, the immune-privilege framework has been substantially revised by advancements in imaging, molecular biology, and clinical research [10, 28]. The current understanding portrays the CNS as an immunologically specialized site that meticulously regulates immune cell trafficking and local inflammation, rather than being entirely isolated from the immune system [16]. Resident cells such as microglia and astrocytes provide intrinsic immune surveillance, while the BBB permits immune cell entry under tightly controlled conditions [3, 63, 64]. The discovery of the glymphatic system and the meningeal lymphatic vessels has unveiled a critical drainage pathway that facilitates waste product clearance and antigen presentation in peripheral lymphoid tissues, bridging local and systemic immune processes [10]. Pathological scenarios vividly illustrate that the CNS can mount or be subjected to immune processes comparable to those in peripheral organs [55]. Moving forward, a progressive understanding of the CNS immune regulation presents opportunities to refine therapeutic interventions for a spectrum of neurological disorders [22]. The challenge lies in modulating inflammatory and reparative processes within the brain to harness the immune system’s protective and clearing abilities while preventing irreparable neural damage [23]. In essence, the once-dominant “immune privilege” paradigm has evolved into an appreciation of the CNS as a site of finely calibrated immunological engagement, participating in a larger, integrated immune system that operates across the entire body [23]. This comprehensive perspective is essential for advancing translational neuroscience and immunology, ultimately enhancing therapeutic strategies for a myriad of CNS pathologies [23].
